# Cardiovascular Risk Scores and Migraine Status

**DOI:** 10.1001/jamanetworkopen.2024.40577

**Published:** 2024-10-22

**Authors:** Linda Al-Hassany, Antoinette MaassenVanDenBrink, Tobias Kurth

**Affiliations:** 1 Division of Vascular Medicine and Pharmacology, Department of Internal Medicine, Erasmus MC, University Medical Center Rotterdam, Rotterdam, the Netherlands; 2 Institute of Public Health, Charité–Universitätsmedizin Berlin, Berlin, Germany

## Abstract

**Importance:**

A previous cohort study in the US found that women with higher cardiovascular risk were more likely to have a history of migraine but less likely to have active migraine. Extrapolating these results to men and European individuals is crucial to understanding the complex association between migraine activity status and vascular health in other populations.

**Objective:**

To evaluate the association pattern between a cardiovascular risk score, the most recent European version of the Systematic Coronary Risk Evaluation 2 (SCORE2) risk estimation system, and migraine activity status in Dutch men and women.

**Design, Setting, and Participants:**

The prospective population-based Lifelines cohort consists of community-dwelling adults residing in the northern part of the Netherlands. Individuals with a terminal illness, incapacitated individuals, including those with a severe mental illness, or who were unable to visit their general practitioner or complete the questionnaires were excluded from participation within Lifelines. Participants whose data on the cardiovascular risk scores and migraine status were complete were included in the analysis. Data on baseline characteristics were collected between November 1, 2006, to December 31, 2014. Cross-sectional and follow-up analyses were conducted within the prospective cohort. Questionnaires were sent approximately every 1.5 to 2.5 years, and the last self-reported migraine assessment took place between October 1, 2019, and January 31, 2021. Data were analyzed from March 1, 2022, to August 16, 2024.

**Exposures:**

The SCORE2 is a sex-specific European cardiovascular risk score that includes age, cholesterol levels, smoking status, diabetes, and systolic blood pressure.

**Main Outcomes and Measures:**

The primary outcome was the association pattern between cardiovascular risk scores and migraine activity status. SCORE2 risk scores were measured once at baseline; groups of the SCORE2 (<1.0%, 1.0% to <2.5%, 2.5% to <5.0%, 5.0% to <7.5%, 7.5% to <10.0%, and ≥10.0%) were created based on the sum of points of individual risk factors. Migraine activity status was assessed using self-reported questionnaires and classified as (1) prevalent (ie, migraine at baseline), (2) incident (ie, no migraine at baseline but migraine in ≥1 follow-up), and (3) none. To evaluate the influence of age, we conducted stratified analyses of the SCORE2 by age categories (<40, 40-49, and ≥50 years).

**Results:**

The total study population consisted of 140 915 individuals at baseline with a mean (SD) age of 44.4 (12.7) years, of whom 58.5% were women. In total, 25 915 individuals (18.4% of the total population) had prevalent migraine and 2224 (1.9% of the 115 000 without prevalent migraine) had incident migraine. The odds of having prevalent and incident migraine, compared with individuals with a SCORE2 category of less than 1.0%, varied and decreased with increasing SCORE2 categories, with odds ratios (ORs) for prevalent migraine ranging from 0.93 (95% CI, 0.90-0.96) for a SCORE2 category of 1.0% to less than 2.5% to 0.43 (95% CI, 0.39-0.48) for a SCORE2 category of at least 10.0% and, for incident migraine, from 0.63 (95% CI, 0.57-0.69) for a SCORE2 category of 1.0% to less than 2.5% to 0.17 (95% CI, 0.10-0.27) for a SCORE2 category of at least 10.0%. A similar pattern was observed in both sexes but more profound in women. In women, ORs for prevalent migraine ranged from 1.21 (95% CI, 1.16-1.25) to 0.70 (95% CI, 0.58-0.83) (vs 1.19 [95% CI, 1.09-1.29] to 0.84 [95% CI, 0.72-0.99] in men) and, for incident migraine, 0.72 (95% CI, 0.64-0.80) to 0.20 (95% CI, 0.07-0.43) (vs 1.18 [95% CI, 0.92-1.52] to 0.44 [95% CI, 0.22-0.78] in men). Models with incident migraine as the outcome showed lower ORs across the ascending cardiovascular risk score categories. Age stratification suggested that the association between cardiovascular risk scores and migraine activity status were unlikely to be strongly influenced by age.

**Conclusions and Relevance:**

In this cohort study of community-dwelling Dutch adults, the odds of having prevalent or incident migraine decreased with increasing cardiovascular risk score categories. These results support the hypothesis that a relatively healthy cardiovascular system increases the probability of having active or developing migraine in the future, especially among women. Sex differences might play a pathophysiological role in the association between migraine activity and vascular health.

## Key Points

**Question:**

What is the association pattern between the Systematic Coronary Risk Evaluation 2 (SCORE2) category and self-reported migraine activity status?

**Findings:**

In a population-based cohort study of 140 915 participants in the Netherlands, the odds of having prevalent migraine and incident migraine in particular decreased with increasing SCORE2 categories in men and women.

**Meaning:**

These results suggest that having active or developing migraine may be associated with a relatively healthy vascular system, as assessed by traditional cardiovascular risk factors, despite migraine being a prominent marker for increased cardiovascular risk.

## Teaser

This cohort study evaluates the association pattern between a cardiovascular risk score and migraine activity status in a large cohort of men and women from the Netherlands.

## Introduction

Migraine is a common neurovascular headache disorder with episodic or chronic forms, characterized by moderate-to-severe headache attacks and accompanying symptoms, including nausea, vomiting, and photophobia and phonophobia.^1^ Approximately one-third of patients with migraine experience (additional) transient neurological disturbances (ie, aura symptoms).^1^ Migraine has been ranked as the first most disabling disorder in women younger than 50 years, in whom the 1-year prevalence is the highest.^2,3^ Besides the direct burden caused by its disabling attacks, migraine headache—especially with aura—is associated with major cardiovascular and cerebrovascular disease events, including ischemic and hemorrhagic stroke, myocardial infarction, and atrial fibrillation.^4,5,6,7^ Although a clinically underappreciated cardiovascular risk factor,^8,9^ migraine is slowly being recognized as such, and has recently been included in a cardiovascular risk prediction score^10^ or added to existing prediction models.^11^

Contradictory findings exist in the literature concerning the association between migraine and markers of cardiovascular risk. While some studies have shown no association—or even an inverse association—between migraine and coronary and cranial calcifications^12,13^ as well as macrovascular atherosclerosis,^14^ these outcomes stand in contrast to other research that has reported elevated markers of atherosclerosis, specifically in individuals with migraine with aura.^15,16^ An overlooked aspect herein may be the migraine activity status and heterogeneity of the manifestation of migraine during a lifetime.^17,18,19^ The association between migraine and cardiovascular disease has been demonstrated to vary by different categories of the Framingham Risk Score (FRS),^7^ an algorithm that estimates the individual 10-year risk of coronary heart disease^20^ and that is considered a marker of atherosclerosis. Results from a cohort study among female health professionals from the US showed that women in higher FRS risk groups had a higher likelihood of having a history of migraine but a lower risk of having active migraine when compared with women without migraine and who were in the lowest FRS category.^21^

These results suggest a complex interplay between migraine activity states and vascular health. However, whether these findings can be extrapolated to men and the European setting remains unclear. Therefore, we aimed to evaluate the association pattern between a cardiovascular risk score, the most recent European version of the Systematic Coronary Risk Evaluation 2 (SCORE2) risk estimation system,^22^ with migraine activity status in a large cohort of men and women from the Netherlands.

## Methods

### Study Population

This study is reported according to the Strengthening the Reporting of Observational Studies in Epidemiology (STROBE) guidelines. All analyses were performed within Lifelines, an ongoing multidisciplinary prospective population-based cohort study, consisting of 3 generations and a total number of 167 729 participants (including approximately 140 000 adults aged 18-65 years and 12 000 aged >65 years), which is 10% of the population living in the northern part of the Netherlands (provinces Groningen, Friesland, and Drenthe). Participants were mainly recruited via general practitioners and were also asked to invite family members. In addition, interested individuals were allowed to register online for participation. No specific inclusion criteria were applied, and the risk of selection bias has been demonstrated to be low.^23^ Individuals with a terminal illness, (incapacitated) individuals with a severe mental illness, and individuals who were unable to (1) visit their general practitioner, (2) fill in questionnaires, or (3) understand the Dutch language were excluded from participation. All participants signed an informed consent form.

Lifelines uses a broad range of investigative procedures in assessing the biomedical, sociodemographic, behavioral, physical, and psychological factors contributing to the health, including aging, and disease of the general population, with a special focus on multimorbidity and complex genetics. The Lifelines Cohort Study is conducted according to the principles of the Declaration of Helsinki and approved by the Medical Ethics Committee of the University Medical Center Groningen, Groningen, the Netherlands. Assessments are performed at the laboratory center of the University Medical Center Groningen, which is certified according to Dutch, European Union, and international standards. The overall design and rationale of this cohort have been described in detail elsewhere.^24,25^

### Study Procedures

The baseline characteristics were collected between November 1, 2006, and December 31, 2014. Follow-up procedures included visits to the research site every 5 years, during which blood samples were collected, among other things. Questionnaires sent approximately every 1.5 to 2.5 years were completed.

All individuals 18 years or older who signed informed consent before participation during the first visit were included in the present study. We studied the SCORE2 categories (collected during baseline) as a predictive factor to estimate whether migraine is present at baseline or will occur during follow-up. Therefore, we excluded participants for whom 1 or multiple variables to calculate the SCORE2 were missing (n = 6458) and/or those whose migraine status (yes or no) was not confirmed during baseline (n = 3499). This yielded a total number of 140 915 individuals for this study.

### SCORE2

The SCORE2 is a sex-specific cardiovascular risk score that is currently recommended by the European guidelines for cardiovascular disease.^26^ It includes age, total cholesterol level, high-density lipoprotein cholesterol level, current smoking status (yes or no), diabetes (yes or no), and systolic blood pressure. The SCORE2 estimates the 10-year risk of first-onset fatal and nonfatal cardiovascular disease.^22^ SCORE2 cardiovascular risk scores were computed for every individual included in the study using baseline characteristics. For the calculation of the SCORE2, we calculated the risk estimates for the Netherlands (ie, a low-risk region).

Age at the time of completion of the baseline questionnaire was used. Sodium fluoride heparin gel tubes were used to collect venipuncture samples in mainly fasting individuals (97.8%) to assess cholesterol levels. Collected blood was transferred to the central laboratory of the University Medical Center Groningen and stored at −80 °C, while routine clinical chemistry assays were performed from other aliquots. Serum levels of total and high-density lipoprotein cholesterol levels were determined on the day of collection using an (enzymatic) colorimetric method, and all lipid levels were measured using a chemistry analyzer (Modular P; Roche). Self-reported questionnaires were used to obtain information about smoking status (ever or current), antihypertensive drug use, and the presence of diabetes. Systolic (and diastolic) blood pressures were automatically measured every minute for 10 minutes using commercially available monitors (DinaMap PRO 100 or 100V2; GE) in a seated position. The mean of the final 3 registered blood pressure measurements was used for further analyses.

In accordance with previous studies, we created groups of the SCORE2 (<1.0%, 1.0% to <2.5%, 2.5% to <5.0%, 5.0% to <7.5%, 7.5% to <10.0%, and ≥10.0%) based on the sum of points of individual risk factors and taking into account that the assigned risk category (low, moderate, and high) depends on the age category (<50 and 50-69 years).^22^ Considering the relatively large number of individuals with a SCORE2 of less than or equal to 2.5%, we further categorized these individuals to identify those with a very low risk (<1.0%).

### Migraine Assessment

Migraine was assessed using self-administered questionnaires. During baseline, the participant was asked whether they have (had) migraine as follows: “Could you indicate which of the following disorders you have (had)?” for which “migraine” was one of the answer options. Subsequently, during 4 follow-up moments, the participant was asked whether migraine had started since the last time they filled in the Lifelines questionnaire. In these follow-up questionnaires, participants were asked: “Did the health problems listed below start since the last time you filled in the Lifelines questionnaire?” and they had to answer “yes” or “no” for migraine. The last migraine assessment took place between October 1, 2019, and January 31, 2021.

Individuals were categorized into (1) those who reported having migraine during baseline (baseline or prevalent migraine), (2) those who did not report having migraine during baseline but who reported having migraine during at least 1 follow-up assessment (incident migraine), and (3) those who did not report having migraine during baseline and who did not report migraine during the follow-up assessment (no migraine). The baseline migraine question does not distinguish participants who only experienced migraine in the past (>1 year prior to inclusion, or history of migraine) from those who only experienced migraine at baseline (active migraine). Therefore, we could not include a fourth separate category consisting of participants with a history of migraine as used in the Women’s Health Study.^21^

### Additional Baseline Characteristics

Racial and ethnic categories (Asian, Black, White Eastern and Western European, White Mediterranean or Arabic, or other [mainly consisting of individuals with multiethnic background]) were based on answers to the question of which of these populations participants considered themselves to belong. The educational attainment score (low, middle, or high) was calculated for adult participants based on self-reported highest obtained degree or diploma. Any use of hormones was a composite variable for women only and based on the self-reported answers to questions on whether hormonal contraception was ever used and whether hormone therapy for menopause was ever received.

Total activity scores of the validated Short Questionnaire to Assess Health-Enhancing Physical Activity questionnaire were used to provide an indication of weekly habitual physical activity.^27,28^ The activity score (total score per week) was calculated by multiplying minutes per week by a factor for intensity.

The Lifelines Diet Score, a relative measure of overall diet quality, was derived from a 110-item food frequency questionnaire and was based on the intake of food groups with positive and negative health effects.^29^ The intake of these food groups was expressed in grams per 1000 kilocalories, with a higher score indicating a higher diet quality.

### Statistical Analysis

Data were analyzed from March 1, 2022, to August 16, 2024. We aimed to study the association pattern of the SCORE2 with migraine status (prevalent migraine or incident migraine). Considering our study in a European population, our primary objective was to investigate the SCORE2. Our secondary objective was to report the association pattern of the SCORE2 in women and men separately.

We calculated means and SDs of continuous variables and frequencies and percentages of categorical baseline characteristics according to the participants’ SCORE2 category. We calculated odds ratios (ORs) with 95% CIs using logistic regression analyses with either prevalent or incident migraine as the outcome to describe the association pattern with SCORE2. Thus, both cross-sectional and follow-up analyses were conducted within this prospective cohort. The reference group included individuals with the lowest SCORE2 category (<1.0%). All models were applied to the total population and were subsequently stratified by sex. To evaluate the influence of age, we conducted stratified analyses of the SCORE 2 by age categories (<40, 40-49, and ≥50 years). Due to a low number of individuals in the groups with SCORE2 of 7.5% to less than 10.0% and at least 10.0%, we merged these highest categories for the age-stratified analyses. In addition to performing stratified analyses for both age categories and sex, we formally tested the significance of an interaction term between similar SCORE2 categories and age categories (<40, 40-49, and ≥50 years) as well as SCORE2 categories and sex. We tested for statistically significant effect modification by comparing 2 models with and without these interaction terms using the likelihood-ratio test.

In a first sensitivity analysis, we presented the age-stratified associations for incident and prevalent migraine based on age categories and corresponding risk classifications (ie, low to moderate, high, and very high risk) defined by the European Society of Cardiology guidelines.^30^ Indeed, it could be argued that the same reference category (ie, <1.0%) is not relevant in all cardiovascular risk categories.

In a second sensitivity analysis, we further evaluated the association between the SCORE2 categories and incident migraine during follow-up using a Cox proportional hazards regression model. If no migraine was reported during the follow-up period, the participant was censored at the date of the last follow-up. In cases where the date of the last follow-up was also unknown, the date of the final assessment (January 2021) was used. The proportionality assumption for the Cox proportional hazards regression model was evaluated via the Schoenfeld residuals test and plot. No violation of the proportional hazards assumption was observed. Additionally, a Kaplan-Meier plot was generated for time to incident migraine, by SCORE2 categories.

We used an a priori level of significance set at *P* ≤ .05, and all hypothesis tests were 2 sided. Because of our clearly defined primary and secondary analyses, we did not adjust *P* values for multiple testing, as our analyses were not exploratory. All statistical analyses were performed using R, version 4.2.0, and RStudio, version 2022.2.0.443 (R Program for Statistical Computing), as well as Stata/MP, version 13.1, for Windows (StataCorp LLC).

## Results

The total study population consisted of 140 915 individuals with a mean (SD) age of 44.4 (12.7) years, 82 372 (58.5%) of whom were women and 58 543 (41.5%) were men. In total, 25 915 individuals (18.4% of the total population) had prevalent migraine and 2224 (1.9% of the 115 000 without prevalent migraine) had incident migraine.

Of the 140 915 individuals included in our study, 49 690 had a SCORE2 of less than 1.0%; 43 343, a SCORE2 of 1.0% to less than 2.5%; 28 993, a SCORE2 of 2.5% to less than 5.0%; 10 835, a SCORE2 of 5.0% to less than 7.5%; 4594, a SCORE2 of 7.5% to less than 10.0%; and 3460, a SCORE2 of at least 10.0% (Table 1). The study population mainly consisted of White European individuals (117 145 [83.1%]); owing to some SCORE2 categories having fewer than 10 participants for some ethnic groups, exact percentages of Asian, Black, and White Mediterranean participants could not be calculated. As expected, cardiovascular determinants, including age, blood pressure, antihypertensive drug use, cholesterol and lipid levels, body mass index, and the prevalence of diabetes and ever or current smoking increased with increasing SCORE2 categories. However, other determinants decreased (eg, the use of hormonal contraception and hormone therapy among women) or remained relatively stable (eg, the Lifelines Diet Score). Most individuals with the lowest SCORE2 category consisted of women (82.0%), with increasing male to female ratios in higher SCORE2 categories. The number of participants with missing information is displayed in eTable 1 in Supplement 1.

**Table 1.** Baseline Characteristics of Lifelines Participants According to SCORE2 Categories

Characteristic	SCORE2 category^a^					
	<1.0% (n = 49 690)	1.0 to <2.5% (n = 43 343)	2.5 to <5.0% (n = 28 993)	5.0 to <7.5% (n = 10 835)	7.5 to <10.0% (n = 4594)	≥10.0% (n = 3460)
Sex, No. (%)						
Female	40 748 (82.0)	24 025 (55.4)	11 699 (40.4)	3662 (33.8)	1379 (30.0)	859 (24.8)
Male	8942 (18.0)	19 318 (44.6)	17 294 (59.6)	7173 (66.2)	3215 (70.0)	2601 (75.2)
Age, mean (SD), y	33.8 (8.5)	43.9 (7.7)	51.4 (8.2)	58.8 (8.4)	64.6 (7.5)	71.0 (7.8)
Race and ethnicity, No. (%)						
Asian	253 (0.5)	153 (0.4)	113 (0.4)	40 (0.4)	23 (0.5)	<10 (<0.3)
Black	80 (0.2)	66 (0.2)	35 (0.1)	10 (0.1)	<10 (<0.2)	<10 (<0.3)
White, European	40 109 (80.7)	35 720 (82.4)	24 627 (84.9)	9479 (87.5)	4091 (89.1)	3119 (90.1)
White, Mediterranean or Arabic	182 (0.4)	128 (0.3)	67 (0.2)	20 (0.2)	<10 (<0.2)	<10 (<0.3)
Other^b^	474 (1.0)	380 (0.9)	215 (0.7)	75 (0.7)	25 (0.5)	19 (0.5)
Educational attainment, No. (%)						
Low	7552 (15.2)	12 320 (28.4)	11 717 (40.4)	5348 (49.4)	2381 (51.8)	1857 (53.7)
Middle	23 048 (46.4)	18 220 (42.0)	9943 (34.3)	2967 (27.4)	1075 (23.4)	708 (20.5)
High	18 767 (37.8)	12 454 (28.7)	6996 (24.1)	2301 (21.2)	984 (21.4)	748 (21.6)
Diastolic blood pressure, mean (SD), mm Hg	69.1 (7.4)	74.1 (8.6)	77.4 (9.3)	78.9 (9.8)	79.3 (10.2)	79.5 (10.4)
Systolic blood pressure, mean (SD), mm Hg	117 (11.0)	125 (12.8)	131 (13.8)	136 (15.1)	142 (16.2)	148 (18.8)
Antihypertensive drug use, No. (%)	900 (1.8)	3020 (7.0)	4145 (14.3%)	2605 (24.0)	1502 (32.7)	1413 (40.8)
BMI, mean (SD)	24.5 (4.1)	26.3 (4.3)	27.1 (4.1)	27.5 (4.0)	27.7 (3.8)	27.7 (3.8)
Waist-to-hip ratio, mean (SD)	0.86 (0.07)	0.91 (0.08)	0.94 (0.08)	0.96 (0.08)	0.97 (0.08)	0.98 (0.07)
Total cholesterol level, mean (SD), mg/dL	177.6 (30.9)	196.9 (34.7)	216.2 (38.6)	216.2 (42.5)	212.4 (46.3)	204.6 (46.3)
HDL cholesterol level, mean (SD), mg/dL	61.8 (15.4)	57.9 (15.4)	54.1 (15.4)	54.1 (15.4)	50.2 (15.4)	50.2 (15.4)
LDL cholesterol level, mean (SD), mg/dL	104.2 (27.0)	127.4 (30.9)	142.9 (34.7)	142,9 (38.6)	139.0 (42.5)	131.3 (42.5)
Triglyceride level, mean (SD), mg/dL	79.6 (35.4)	106.2 (53.16)	132.7 (79.6)	141.6 (106.2)	141.6 (106.2)	141.6 (123.9)
Diabetes, No. (%)	71 (0.1)	306 (0.7)	771 (2.7)	762 (7.0)	612 (13.3)	843 (24.4)
Women who ever used hormonal contraception and/or hormone therapy, No. (%)	34 621 (85.0)	19 781 (82.3)	9496 (81.2)	2828 (77.2)	957 (69.4)	475 (55.3)
Smoking status, No. (%)						
Ever	17 683 (35.6)	23 987 (55.3)	19 462 (67.1)	7829 (72.3)	3402 (74.1)	2630 (76.0)
Current	5266 (10.6)	9760 (22.5)	9030 (31.1)	3632 (33.5)	1329 (28.9)	944 (27.3)
SQUASH activity score, mean (SD)^c^	7092 (4004)	7533 (4364)	8053 (5027)	8487 (5452)	8074 (5096)	7484 (5036)
Lifelines diet score, mean (SD)^d^	23.4 (6.1)	24.0 (6.0)	24.3 (6.1)	24.9 (6.0)	25.3 (5.9)	25.2 (5.8)

Abbreviations: BMI, body mass index (calculated as the weight in kilograms divided by the height in meters squared); HDL, high-density lipoprotein; LDL, low-density lipoprotein; SCORE2, Systematic Coronary Risk Evaluation 2; SQUASH, Short Questionnaire to Assess Health-Enhancing Physical Activity.

SI conversion factors: To convert cholesterol to mmol/L, multiply by 0.0259; triglycerides to mmol/L, multiply by 0.0113.

^a^ Not all variables total 100% due to missing values, as specified in eTable 1 in Supplement 1. Frequencies of less than 10 are displayed as such to prevent traceability.

^b^ Mainly reflects individuals with multiethnic background.

^c^ Calculated by multiplying minutes per week by a factor for intensity, with higher scores indicating a higher level of physical activity.

^d^ Calculated from quintiles of intake of positive and negative food groups in grams per 1000 kcal, with a higher score indicating better quality diet.

In Table 2, the distribution of the absolute and relative frequencies of individuals with and without prevalent migraine and individuals with and without incident migraine are displayed across all SCORE2 categories, indicating that most patients in all 4 migraine groups had a SCORE2 of less than 1.0%. In eTable 2 in Supplement 1, a similar overview is provided but stratified for sex, indicating that most women with prevalent and incident migraine had a SCORE2 of less than 1.0%, while most men with prevalent and incident migraine had a SCORE2 of 1.0% to less than 2.5%.

**Table 2.** Individuals With and Without Migraine Before and During Baseline (Prevalent Migraine) and During Follow-Up (Incident Migraine) According to SCORE2 Categories^a^

SCORE2 category	Prevalent migraine, No. (%)		Incident migraine, No. (%)	
	Yes (n = 25 915)	No (n = 115 000)	Yes (n = 2224)	No (n = 112 776)
<1.0%	10 237 (39.5)	39 453 (34.3)	1167 (52.5)	38 286 (33.9)
1.0% to <2.5%	8428 (32.5)	34 915 (30.4)	654 (29.4)	34 261 (30.4)
2.5% to <5.0%	4795 (18.5)	24 198 (21.0)	290 (13.0)	23 908 (21.2)
5.0% to <7.5%	1573 (6.1)	9262 (8.1)	66 (3.0)	9196 (8.2)
7.5% to <10.0%	533 (2.1)	4061 (3.5)	31 (1.4)	4030 (3.6)
≥10.0%	349 (1.3)	3111 (2.7)	16 (0.7)	3095 (2.7)

Abbreviation: SCORE2, Systematic Coronary Risk Evaluation 2.

^a^ Percentages are expressed as proportions of the total population with and without prevalent migraine (n = 140 915) and proportions of the total population with and without incident migraine (total n = 115 000).

The results of the logistic regression models are presented in Table 3, indicating that the odds of having prevalent migraine (at baseline or in the past) when compared with the reference group, that is, individuals with a SCORE2 category of less than 1.0%, varied and decreased with increasing SCORE2 categories. Compared with individuals with a SCORE2 category of less than 1.0%, individuals with prevalent migraine had an OR of 0.93 (95% CI, 0.90-0.96) for a SCORE2 category of 1.0% to less than 2.5%, 0.76 (95% CI, 0.74-0.79) for a SCORE2 category of 2.5% to less than 5.0%, 0.65 (95% CI, 0.62-0.69) for a SCORE2 category of 5.0% to less than 7.5%, 0.51 (95% CI, 0.46-0.55) for a SCORE2 category of 7.5% to less than 10.0%, and 0.43 (95% CI, 0.39-0.48) for a SCORE2 category of at least 10.0%. A similar pattern was observed in both women and men and for incident migraine as the outcome. In women, ORs for prevalent migraine ranged from 1.21 (95% CI, 1.16-1.25) to 0.70 (95% CI, 0.58-0.83) (vs 1.19 [95% CI, 1.09-1.29] to 0.84 [95% CI, 0.72-0.99] in men) and, for incident migraine, 0.72 (95% CI, 0.64-0.80) to 0.20 (95% CI, 0.07-0.43) (vs 1.18 [95% CI, 0.92-1.52] to 0.44 [95% CI, 0.22-0.78] in men) (Table 3).

**Table 3.** Associations of the Categorical SCORE2 With Prevalent and Incident Migraine in the Total Lifelines Population and Stratified by Sex^a^

SCORE2 category	OR (95% CI)	
**Prevalent migraine** ^b^	**Incident migraine** ^c^
**Total population**		
<1.0%	1 [Reference]	1 [Reference]
1.0% to <2.5%	0.93 (0.90-0.96)	0.63 (0.57-0.69)
2.5% to <5.0%	0.76 (0.74-0.79)	0.40 (0.35-0.45)
5.0% to <7.5%	0.65 (0.62-0.69)	0.24 (0.18-0.30)
7.5% to <10.0%	0.51 (0.46-0.55)	0.25 (0.17-0.35)
≥10.0%	0.43 (0.39-0.48)	0.17 (0.10-0.27)
**Women**		
<1.0%	1 [Reference]	1 [Reference]
1.0% to <2.5%	1.21 (1.16-1.25)	0.72 (0.64-0.80)
2.5% to <5.0%	1.07 (1.01-1.12)	0.39 (0.32-0.47)
5.0% to <7.5%	0.97 (0.90-1.05)	0.28 (0.19-0.40)
7.5% to <10.0%	0.80 (0.70-0.92)	0.33 (0.18-0.55)
≥10.0%	0.70 (0.58-0.83)	0.20 (0.07-0.43)
**Men**		
<1.0%	1 [Reference]	1 [Reference]
1.0% to <2.5%	1.19 (1.09-1.29)	1.18 (0.92-1.52)
2.5% to <5.0%	1.29 (1.18-1.41)	1.06 (0.81-1.38)
5.0% to <7.5%	1.17 (1.05-1.30)	0.56 (0.38-0.82)
7.5% to <10.0%	0.91 (0.78-1.05)	0.58 (0.34-0.94)
≥10.0%	0.84 (0.72-0.99)	0.44 (0.22-0.78)

Abbreviations: OR, odds ratio; SCORE2, Systematic Coronary Risk Evaluation 2.

^a^ Includes the total population with and without prevalent migraine (n = 140 915) and the total population with and without incident migraine (n = 115 000).

^b^ A significant interaction was observed between sex and SCORE2 categories for prevalent migraine (*P* < .001).

^c^ A significant interaction was observed between sex and SCORE2 categories for incident migraine (*P* < .001).

Notably, logistic models with incident migraine as the outcome displayed lower ORs across the ascending SCORE2 categories compared with models with prevalent migraine as the outcome, ranging from 0.63 (95% CI, 0.57-0.69) for a SCORE2 category of 1.0% to less than 2.5% to an OR of 0.17 (95% CI, 0.10-0.27) for a SCORE2 category of at least 10.0% (*P* < .001 for interaction). A significant interaction was also observed between sex and SCORE2 categories for both incident and prevalent migraine (*P* < .001 for both interactions; Table 3). As presented in Table 4, age stratification indicated that ORs of the SCORE2 categories for both prevalent and incident migraine did not show major differences, implying that our results are unlikely to be strongly influenced by age. A significant interaction was observed between age and SCORE2 categories for prevalent migraine (*P* < .001), but not for incident migraine (*P* = .29) (Table 4). Our first sensitivity analyses using age categories based on risk classifications (low to moderate, high, and very high risk) similarly revealed no association with age (eTable 3 in Supplement 1).

**Table 4.** Associations of the Categorical SCORE2 With Prevalent and Incident Migraine in the Total Lifelines Population Stratified by Age Categories^a^

SCORE2 category	OR (95% CI)					
	Prevalent migraine^b^			Incident migraine^c^		
	<40 y (n = 48 323)	40-49 y (n = 48 877)	≥50 y (n = 43 715)	<40 y (n = 33 901)	40-49 y (n = 39 095)	≥50 y (n = 36 004)
<1.0%	1 [Reference]	1 [Reference]	1 [Reference]	1 [Reference]	1 [Reference]	1 [Reference]
1.0% to <2.5%	0.71 (0.67-0.75)	0.76 (0.72-0.80)	1.01 (0.84-1.21)	0.55 (0.46-0.65)	0.65 (0.56-0.75)	0.64 (0.38-1.16)
2.5% to <5.0%	0.59 (0.51-0.67)	0.54 (0.51-0.58)	0.69 (0.58-0.83)	0.40 (0.25-0.60)	0.47 (0.38-0.57)	0.36 (0.21-0.65)
5.0% to <7.5%	0.96 (0.64-1.40)	0.43 (0.37-0.50)	0.55 (0.46-0.66)	0.24 (0.01-1.09)	0.35 (0.21-0.55)	0.23 (0.13-0.43)
≥7.5%	Too few participants	0.34 (0.24-0.49)	0.39 (0.32-0.47)	2.41 (0.13-12.00)	0.32 (0.078-0.84)	0.22 (0.13-0.43)

Abbreviations: OR, odds ratio; SCORE2, Systematic Coronary Risk Evaluation 2.

^a^ Includes the total population with and without prevalent migraine (n = 140 915) and the total population with and without incident migraine (n = 115 000).

^b^ In the total population, a significant interaction was observed between age categories and SCORE2 categories for prevalent migraine (*P* < .001).

^c^ In the total population, no significant interaction was observed between age categories and SCORE2 categories for incident migraine (*P* = .29).

Further, in a second sensitivity analysis using Cox proportional hazards regression models to estimate the association between SCORE2 categories and incident migraine, while accounting for the time to the event, we found similar estimates as those obtained from the logistic regression models. Compared with individuals with a SCORE2 category of less than 1.0%, the hazard ratios for incident migraine were 0.63 (95% CI, 0.58-0.70) for a SCORE2 category of 1.0% to less than 2.5%, 0.47 (95% CI, 0.41-0.54) for a SCORE2 category of 2.5% to less than 5.0%, 0.33 (95% CI, 0.26-0.43) for a SCORE2 category of 5.0% to less than 7.5%, 0.40 (95% CI, 0.28-0.57) for a SCORE2 category of 7.5% to less than 10.0%, and 0.28 (95% CI, 0.17-0.46) for a SCORE2 category of at least 10.0%. Kaplan-Meier estimates with 95% CIs are presented in the Figure.

**Figure.**
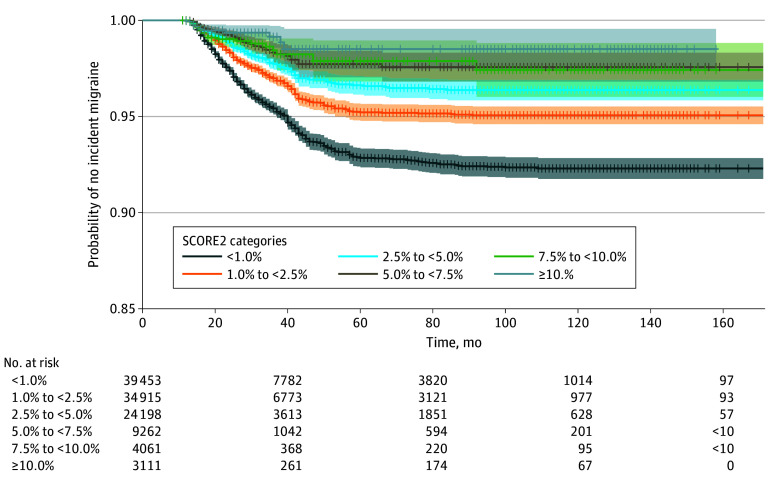
Cumulative Estimates of the Probability of Not Having an Incident Migraine These estimates are stratified by the Systematic Coronary Risk Evaluation 2 (SCORE2) risk category. SCORE2 risk categories range from less than 1.0% (lowest risk) to at least 10.0% (highest risk). Shaded areas represent 95% CIs.

## Discussion

In the population-based and apparently healthy Dutch Lifelines cohort, we examined the association pattern of the European cardiovascular risk score, the SCORE2, and prevalent as well as incident migraine in the predictive domain. For the SCORE2, we found that in general, individuals with prevalent migraine, but especially those with incident migraine, were less likely to be in the SCORE2 categories of 1.0% to less than 2.5%, 2.5% to less than 5.0%, 5.0% to less than 7.5%, 7.5% to less than 10.0%, and at least 10.0% compared with individuals having an SCORE2 category of less than 1.0%. Notably, the odds of incident migraine decreased with increasing SCORE2 categories compared with individuals without migraine, and this finding is substantiated by our sensitivity analyses using the Cox proportional hazards model. The decreased association with increasing cardiovascular risk score categories was observed in both men and women, indicating that the underlying vascular health status is a predictor of having or developing migraine. Indeed, our results support the hypothesis that a relatively healthy cardiovascular system (expressed as a lower SCORE2 category) increases the probability of developing migraine in the future (incident migraine). The potentially younger age of patients with incident migraine does not fully explain this association, as indicated by our age-stratified analyses of the SCORE2. Further, the ORs point in the same direction in both women and men, but the association of both risk scores is stronger in women.

A previous study showed a doubling in the odds of having an elevated FRS (for myocardial infarction or coronary heart disease death) for individuals with current migraine with aura compared with individuals without migraine, but not in individuals with migraine without aura.^31^ This is in contrast to the results of other population-based research showing increased risks in people with migraine both with and without aura.^32^ However, in elderly people with migraine, a worse cardiovascular risk profile was not observed.^33^ These discrepancies in the literature indicate that the association between cardiovascular risk scores, as preclinical measures of atherosclerosis,^34,35,36^ might be attributable to differences in age categories and migraine activity (past, active, or incident) of the study populations.

Our results build upon previous work,^21^ confirming a crucial uncertainty that the observed associations, especially in women, are not attributable only to the influence of age on migraine status. In addition, it supports our hypothesis that the SCORE2, showing an inverse association for incident migraine in particular, might not fully capture the elevated cardiovascular risk associated with migraine. Indeed, the SCORE2 is rather a determinant of macrovascular disease composed of traditional risk factors that do not necessarily reflect microvascular pathology or nontraditional mechanisms that may underlie the increased cardiovascular risk of migraine. Research findings support the hypothesis that migraine is associated with retinopathy, indicating that retinal microvascular abnormalities reflect the condition of the microvasculature.^37^ Additionally, these studies found no excess and even a reduced prevalence of large-artery atherosclerosis, even among individuals who have experienced a previous ischemic stroke.^38,39^ These nontraditional mechanisms may be associated with (1) endothelial dysfunction and decreased bioavailability of nitric oxide as an early step of atherosclerosis^40,41^; (2) an altered activity of (vasodilating) neuropeptides, including calcitonin gene–related peptide activity,^42^ which exerts a pivotal role in migraine pathophysiology^43^ and a potential protective role in atherosclerosis due to positive actions on vessel remodeling, as demonstrated in rat studies^44^; (3) the activation of transient vanilloid receptor potential type 1 channels exhibiting a protective effect against atherosclerosis, probably via calcitonin gene–related peptide release and modulation of the nitric oxide pathway^45,46^; and (4) hypercoagulability and microembolism that may be linked to altered neuronal activity, including cortical spreading depression, in migraine.^47^ Our observations of decreasing ORs of having prevalent and incident migraine with increasing SCORE2 categories might indicate that individuals whose cardiovascular system is more deteriorated due to arterial stiffness have a lower vasodilatory capacity of the meningeal and cerebral arteries to have or develop (future) migraine. However, it should be acknowledged that this view would ignore nonvascular mechanisms involved in migraine pathophysiology, including the central process of cortical spreading depolarization that underlines migraine auras.^48^

In young adults, the SCORE appears to be more accurate at predicting low brachial artery flow-mediated dilatation, a measure of endothelial dysfunction.^49,50^ However, no previous studies focused on the association between SCORE2 and migraine.

Further, in women, the association of SCORE2 was more profound and less likely to be influenced by age when compared with men. Given the known changes in migraine presentation over time, it is feasible that biological sex modifies the effect of migraine on cardiovascular risk.^51,52^ This might be explained by hormonal and nonhormonal influences on the cardiovascular system and sex differences in cardiac and vascular aging.^53^ Also, sex and gender differences in migraine pathophysiology might be an explanation, including hormonal influences (such as effects of estrogen and oxytocin) and differences in neuron activity and brain structures.^51,54,55,56^ Another explanation might relate to age-dependent sex differences in cardiometabolic risk factors, including cholesterol levels, that were observed in this cohort.^57^ We also observed a higher proportion of women using hormonal contraception or therapy in the lower SCORE2 risk categories, likely due to several factors: (1) increased hormone use among younger, premenopausal women^58^; (2) earlier initiation of hormonal contraceptives in younger generations; (3) the elevated cardiovascular risk of combined oral contraceptives, contraindicating their use in women at high risk^59^; and (4) recall bias in older women. Age did not change the association between SCORE2 and migraine status in a meaningful way, suggesting generational differences in hormone use. Future studies should examine the independent influence of hormonal treatments on the link between cardiovascular risk and migraine, considering their role in hypercoagulability^60^ and cerebrovascular risk.^47^

Notably, differences between the overall and sex-stratified ORs were observed, for prevalent migraine and lower SCORE2 categories in particular. We hypothesize that this difference could be a manifestation of the noncollapsibility of the OR, a statistical artifact.^61,62^

### Limitations and Strengths

Several limitations should be taken into account when interpreting our results. First, migraine was self-reported and not based on the *International Classification of Headache Disorders* criteria, potentially resulting in misclassification. While an excellent agreement between self-reported migraine and the *International Classification of Headache Disorders* criteria has been previously demonstrated in the Women’s Health Study,^63^ we cannot exclude the potential misclassification of migraine in the present cohort. Indeed, participants in the Women’s Health Study are health care professionals, who might be more knowledgeable about and aware of the clinical presentation or diagnosis of migraine, and therefore, these results might not be applicable to the Lifelines cohort. Further, in elderly patients, migraine aura without headache might not be directly recognized as migraine. Second, as no specific questions on aura symptoms were asked, we could not distinguish migraine with aura from migraine without aura, ignoring the biological differences between the 2 migraine manifestations^64^ and their differential association with cardiovascular disease.^65^ Third, in contrast to a previous study, we could not distinguish between a history of migraine and active migraine.^21^ Fourth, as expected, the number of participants with incident migraine is relatively lower than those with prevalent migraine, reducing the statistical power of the analyses in this group. Fifth, it is important to mention that the SCORE2 was primarily validated in individuals 40 years and older, which makes our estimates in the younger population less certain. Last, while the racial and ethnic distribution of the Lifelines cohort is roughly similar to that of the Women’s Health Study (which includes approximately 95% White participants), it limits the generalizability of our findings to other populations.

Notwithstanding these limitations, the large number of participants and availability of longitudinal data on the course of migraine allowed us to assess the association patterns between cardiovascular risk scores and migraine. Further, we were able to stratify by sex, adding particular evidence of the association between cardiovascular risk and migraine among men. Further studies should focus on identifying the underlying, possible nonatherosclerotic pathophysiological mechanisms and biomarkers reflecting microvascular dysfunction to improve the prediction of cardiovascular end points in patients with migraine.

## Conclusions

Analyses of the association pattern of the SCORE2 with migraine status in a large population-based Dutch cohort showed that, especially in women, the odds of having prevalent or incident migraine decreased with increasing risk score categories. These results suggest that having or getting migraine may be associated with a relatively healthy vascular system, as assessed by traditional cardiovascular risk factors, despite migraine being a prominent marker for cardiovascular risk.

## Notes

Supplement 1. **eTable 1.** Number of Missing Baseline Characteristics of Lifelines Participants According to SCORE2 Categories **eTable 2.** Number of Women and Men With and Without Migraine Before and During Baseline (Prevalent Migraine) and During Follow-Up According to SCORE2 Categories **eTable 3.** Associations of the Categorical SCORE2, Based on Risk Categories in Primary Prevention Defined by the European Society of Cardiology, With Prevalent and Incident Migraine in the Lifelines Population 40 Years and Older, Stratified by Age Categories

Supplement 2.
Data Sharing Statement
