# Supplementary material for: Cardiovascular Risk Scores and Migraine Status
Source: JAMA Netw Open. 2024 Oct 22;7(10):e2440577. doi: 10.1001/jamanetworkopen.2024.40577 (PMC11581481; doi:10.1001/jamanetworkopen.2024.40577)
Supplement: Supplement 1. — eTable 1. Number of Missing Baseline Characteristics of Lifelines Participants According to SCORE2 Categories eTable 2. Number of Women and Men With and Without Migraine Before and During Baseline (Prevalent Migraine) and During Follow-Up According to SCORE2 Categories eTable 3. Associations of the Categorical SCORE2, Based on Risk Categories in Primary Prevention Defined by the European Society of Cardiology, With Prevalent and Incident Migraine in the Lifelines Population 40 Years and Older, Stratified by Age Categories [file jamanetwopen-e2440577-s001.pdf]

Supplementary Online Content

Al-Hassany L, MaassenVanDenBrink A, Kurth T. Cardiovascular risk scores and migraine status. *JAMA Netw Open*. 2024;7(10):e2440577. doi:10.1001/jamanetworkopen.2024.40577

eTable 1. Number of Missing Baseline Characteristics of Lifelines Participants According to SCORE2 Categories

eTable 2. Number of Women and Men With and Without Migraine Before and During Baseline (Prevalent Migraine) and During Follow-Up According to SCORE2 Categories

eTable 3. Associations of the Categorical SCORE2, Based on Risk Categories in Primary Prevention Defined by the European Society of Cardiology, With Prevalent and Incident Migraine in the Lifelines Population 40 Years and Older, Stratified by Age Categories

This supplementary material has been provided by the authors to give readers additional information about their work.

© 2024 Al-Hassany L et al. *JAMA Network Open*.

eTable 1. Number of Missing Baseline Characteristics of Lifelines Participants According to SCORE2 Categories

	SCORE2 <1%	SCORE2 ≥1 – <2.5%	SCORE2 ≥2.5 – <5%	SCORE2 ≥5 – <7.5%	SCORE2 ≥7.5 – <10%	SCORE2 ≥10%
**Ethnicity**	8592 (17.3%)	6896 (15.9%)	3936 (13.6%)	1211 (11.2%)	444 (9.7%)	307 (8.9%)
**Educational attainment**	323 (0.7%)	349 (0.8%)	337 (1.2%)	219 (2.0%)	154 (3.4%)	147 (4.2%)
**Body mass index** [kg/m^2]	<10 (<0.02%)	15 (0.03%)	<10 (<0.03%)	<10 (<0.09%)	<10 (<0.2%)	<10 (<0.3%)
**Waist-to-hip ratio**	10 (0.02%)	16 (0.04%)	<10 (<0.03%)	<10 (<0.09%)	<10 (<0.2%)	<10 (<0.3%)
**LDL-cholesterol** [mmol/L]	<10 (<0.02%)	0 (0%)	<10 (<0.03%)	<10 (<0.09%)	<10 (<0.2%)	<10 (<0.3%)
**Ever smoker**	202 (0.4%)	153 (0.4%)	68 (0.2%)	21 (0.2%)	<10 (<0.2%)	13 (0.4%)
**SQUASH activity score**	3802 (7.7%)	3119 (7.2%)	2065 (7.1%)	822 (7.6%)	369 (8.0)%	300 (8.7%)
**Lifelines diet score**	5349 (10.8%)	5404 (12.5%)	2849 (9.8%)	671 (6.2%)	150 (3.3%)	63 (1.8%)

Frequencies below 10 are displayed as '<10' to prevent traceability.

© 2024 Al-Hassany L et al. *JAMA Network Open*.

eTable 2. Number of Women and Men With and Without Migraine Before and During Baseline (Prevalent Migraine) and During Follow-Up According to SCORE2 Categories

	PREVALENT MIGRAINE				INCIDENT MIGRAINE			
	**Prevalent migraine (*n = 25915*)**		**No prevalent migraine (*n = 115000*)**		**Incident migraine (*n = 2224*)**		**No incident migraine (*n = 112776*)**	
	Women (n = 19929)	***Men (n = 5986)***	***Women (n = 62443)***	***Men (n = 52557)***	***Women (n = 1691)***	***Men (n = 533)***	***Women (n = 60752)***	***Men (n = 52024)***
**SCORE2 <1%**	9434 (47.3%)	803 (13.4%)	31314 (50.1%)	8139 (15.5%)	1082 (64.0%)	85 (15.9%)	30232 (49.8%)	8054 (15.5%)
**SCORE2 ≥1 – <2.5%**	6405 (32.1%)	2023 (33.8%)	17620 (28.2%)	17295 (32.9%)	442 (26.1%)	212 (39.8%)	17178 (28.3%)	17083 (32.8%)
**SCORE2 ≥2.5 – <5%**	2842 (14.3%)	1953 (32.6%)	8857 (14.2%)	15341 (29.2%)	121 (7.2%)	169 (31.7%)	8736 (14.4%)	15172 (29.2%)
**SCORE2 ≥5 – <7.5%**	830 (4.2%)	743 (12.4%)	2832 (4.5%)	6430 (12.2%)	28 (1.7%)	38 (7.1%)	2804 (4.6%)	6392 (12.3%)
**SCORE2 ≥7.5 – <10%**	269 (1.3%)	264 (4.4%)	1110 (1.8%)	2951 (5.6%)	<14 (<0.8%)	18 (3.4%)	1097 (1.8%)	2933 (5.6%)
**SCORE2 ≥10%**	149 (0.7%)	200 (3.3%)	710 (1.1%)	2401 (4.6%)	<10 (<0.6%)	11 (2.1%)	705 (1.2%)	2390 (4.6%)

Percentages are expressed as proportions of the total number of women or men with and without prevalent migraine (total n = 140,915) and proportions of the total number of women or men with and without incident migraine (total n = 115,000). Frequencies below 10 are displayed as '<10' to prevent traceability.

© 2024 Al-Hassany L et al. *JAMA Network Open*.

eTable 3. Associations of the Categorical SCORE2, Based on Risk Categories in Primary Prevention Defined by the European Society of Cardiology, With Prevalent and Incident Migraine in the Lifelines Population 40 Years and Older, Stratified by Age Categories

	**PREVALENT MIGRAINE**			**INCIDENT MIGRAINE**		
	**OR (95% CI)**			**OR (95% CI)**		
	40-49 years (n = 48877)	***50-69 years (n = 39648)***	***≥70 years (n = 4067)***	***40-49 years (n = 39095)***	***50-69 years (n = 32495)***	***≥70 years (n = 3509)***
**Low to moderate risk**	*reference*	*reference*	*reference*	*reference*	*reference*	*reference*
**High risk**	0.62 (0.59–0.66)	0.60 (0.56–0.63)	0.58 (0.46–0.72)	0.58 (0.48–0.69)	0.49 (0.37–0.63)	0.54 (0.22–1.52)
**Very high risk**	0.41 (0.28–0.57)	0.38 (0.31–0.47)	0.39 (0.27–0.54)	0.41 (0.10–1.07)	0.35 (0.12–0.75)	0.17 (0.01–1.00)

For individuals aged **40-49 years**, the risk categories are as follows: i) Low to moderate risk: <2.5%; ii) High risk: 2.5% to <7.5%; iii) Very high risk: ≥7.5%

For individuals aged **50-69 years**, the risk categories are: i) Low to moderate risk: < 5%; ii) High risk: 5% to <10%; iii) Very high risk: ≥10%

For individuals aged **70 years and older**, the risk categories are: i) Low to moderate risk: < 7.5%; ii) High risk: 7.5% to <15%; iii) Very high risk: ≥15%

Abbreviations: OR = odds ratio; CI = confidence interval

© 2024 Al-Hassany L et al. *JAMA Network Open*.

1. Rapid, online self-assessment of individual risk for cardiovascular events in“ apparently healthy” persons according to the new ESC Guidelines. Accessed August 11, 2024. https://sigmund-silber.com/cms/media/varia/22-08-10_ESC_e-Journal_of_Cardiology_Practice_-_Rapid_online_self-assessment.pdf

© 2024 Al-Hassany L et al. *JAMA Network Open*.
