# Supplementary material for: Cardiovascular Risk Scores and Migraine Status
Source: JAMA Netw Open. 2024 Oct 22;7(10):e2440577. doi: 10.1001/jamanetworkopen.2024.40577 (PMC11581481; doi:10.1001/jamanetworkopen.2024.40577)
Supplement: Supplement 2. — Data Sharing Statement [file jamanetwopen-e2440577-s002.pdf]

Data Sharing Statement

Al-Hassany. Cardiovascular Risk Scores and Migraine Status. *JAMA Netw Open*. Published October 22, 2024. doi:10.1001/jamanetworkopen.2024.40577

Data
Data available: No

Additional Information
Explanation for why data not available: Data may be obtained from a third party and are not publicly available. Researchers can apply to use the Lifelines data used in this study. More information about how to request Lifelines data and the conditions of use can be found on their website (https://www.lifelines.nl/researcher/how-to-apply).
